# Construction, Deployment, and Usage of the Human Reference Atlas Knowledge Graph

**DOI:** 10.1038/s41597-025-05183-6

**Published:** 2025-07-01

**Authors:** Andreas Bueckle, Bruce W. Herr, Josef Hardi, Ellen M. Quardokus, Mark A. Musen, Katy Börner

**Affiliations:** 1 Department of Intelligent Systems Engineering, Luddy School of Informatics, Computing, and Engineering, Indiana University, Bloomington, IN USA; 2 Stanford Center for Biomedical Informatics Research, Stanford University, Stanford, CA USA

**Keywords:** Software, Data integration, Data processing, Data publication and archiving, Databases

## Abstract

The Human Reference Atlas (HRA) for the healthy, adult body is being developed by a team of international, interdisciplinary experts across 25+ consortia. It provides standard terminologies and data structures for describing specimens, biological structures, and spatial positions of experimental datasets and ontology-linked reference anatomical structures (ASs), cell types (CTs), and biomarkers (Bs). This paper introduces the HRA Knowledge Graph (KG) as central data resource for the HRA, supporting cross-scale, biological queries to Resource Description Framework graphs using SPARQL. In May 2025, the HRA KG v2.2 covers 71 organs with 5,800 ASs, 2,268 CTs, 2,531 Bs; it has 10,064,033 nodes, 171,250,177 edges, and a size of 125.84 GB. The HRA KG comprises 13 types of Digital Objects (DOs) using the Common Coordinate Framework Ontology to standardize core concepts and relationships across DOs. This work (1) provides data and code for HRA KG construction; (2) details HRA KG deployment as Linked Open Data; and (3) illustrates HRA KG usage via application programming interfaces, user interfaces, and data products. A companion website is at cns-iu.github.io/hra-kg-supporting-information.

## Background & Summary

The multimodal, three-dimensional (3D) Human Reference Atlas (HRA)^1,2^ aims to map the healthy, adult human body across scales—from the whole body to the single cell and biomarker levels. Data from different sources (organs, technologies, and labs), analyzed and used following standard operating procedures (SOPs, humanatlas.io/standard-operating-procedures), need to be integrated so they can be explored across scales. The HRA Knowledge Graph (KG) defines and provides the core data structures that are used to store, link, query, and explore HRA data.

KGs are widely used to store and interlink data about relevant entities within a specific domain or task. The Google Knowledge Graph^3^ supports Google Search with its billions of searches processed daily. Major online shopping retailers such as Amazon^4^ use KGs to organize products, searches, and media items^5^. KGs across domains are structured using vocabularies, e.g., Friend of a Friend^6^, Simple Knowledge Organization System (SKOS)^7^, and Music Ontology^8,9^. Plus, there exist collaborative efforts for publishing structured data on the web. A major, widely used, light-weight data format for KGs to provide structured data on the web is JavaScript Object Notation for Linked Data (JSON-LD, json-ld.org). Many biomedical ontologies and metadata standards are provided in JSON-LD, such as in the Open Biological and Biomedical Ontologies (OBO) Foundry^10,11^ and National Center for Biomedical Ontology (NCBO) BioPortal^12^, and tools exist to convert ontologies to JSON-LD, such as Protégé (protege.stanford.edu)^13^, ROBOT^14^, and rdflib (rdflib.readthedocs.io/en/stable). Spearheaded by major industry companies, including Google and Microsoft, metadata schemas on schema.org promote the structured representation for data on the web and provide shared vocabulary in various encodings, including Resource Description Framework (RDF, see Box 1) and JSON-LD. They describe entities and relationships in the semantic web and other structured data efforts. An overview of other commonly used vocabularies is available on www.w3.org/wiki/TaskForces/CommunityProjects/LinkingOpenData/CommonVocabularies.

This paper presents the HRA KG v2.2, which uses 10 ontologies (see **Other Ontologies** in **Methods**) to interlink 33 anatomical structures (ASs), cell types (CTs), plus biomarkers (B) tables (see Box 1), 71 3D reference objects for organs, 22 Functional Tissue Units (FTUs)^15^, 11,698 single-cell (sc) datasets, and other HRA Digital Objects (DOs). The HRA KG is accessible via (1) dynamic queries through its SPARQL (see Box 1) endpoint at lod.humanatlas.io/sparql, (2) the Representational State Transfer (REST)-ful HRA application programming interface (API) with clients available in JavaScript, TypeScript, Angular 17+ , and Python 3.6+ (humanatlas.io/api), and (3) several interactive user interfaces (UIs). This work presents open code and infrastructure to construct the HRA KG out of disparate data across tabular/non-tabular and nested/flat HRA DOs while ensuring processed data conforms to 5-star Linked Open Data (LOD)^16^ principles (see Box 1). The HRA KG data can also be accessed via content negotiation from lod.humanatlas.io. Any version of any HRA DO can be obtained in the supported RDF format hosted by the HRA KG.

Box 1 Key terminology used for constructing, deploying, and using the HRA KG **• 3D reference objects:** Mesh-based 3D models describing organs in the male and female body of the HRA. They are used in HRA applications to register and explore tissue blocks as well as associated datasets^55^. All 71 3D reference objects of HRA v2.2 have crosswalks that link their 1,224 3D ASs to ontology terms in Uberon^18^ or Foundational Model of Anatomy (FMA)^53,54^.

**• Anatomical structures, cell types, plus biomarkers tables (ASCT+B):** ASCT+B tables are authored by multiple experts across many consortia. They capture the relationship between ASs (and the ASs located in them), CTs found inside these ASs, and the Bs (genes, proteins) used to characterize the CTs, see details in related publications^1,2^.

**• Cell Type Annotation (CTann):** Azimuth^49^, CellTypist^50,51^, and popV^52^ are used to assign CTs to cells from single-cell/single-nucleus (sc/sn) RNA-seq studies. Crosswalks compiled by experts are used to assign ontology IDs to CTann CTs, see details in a related publication^2^.

**• Common Coordinate Framework (CCF) Ontology:** The CCF Ontology^23^ provides the main vocabulary for constructing atlases of the human body, including the HRA. Critically, the CCF provides a framework for constructing atlases, but is not an atlas itself. It includes concepts and properties needed to describe the human body, from organs down to CTs and Bs, for organizing spatial data, and for capturing donor, sample and dataset metadata published in the HRA KG.

**• Content negotiation:** This mechanism is used by Hypertext Transfer Protocol (HTTP) servers to deliver different versions of a resource at the same Uniform Resource Identifier (URI) based on the parameters given in the HTTP request (developer.mozilla.org/en-US/docs/Web/HTTP/Content_negotiation). HTTP requests can specify headers which provide additional information for the server to act on. Using the *Accept* header, an agent can specify what format (or a ranked list of acceptable formats) they would like the response returned in. A web browser will typically request *text/html*, but machines or programmers may request other formats like *application/json* or any of the RDF formats supported by the HRA KG.

**• Crosswalks:** A matching of terms in HRA DOs to ontology terms in the ASCT+B tables^1^. Crosswalks can link, e.g., 2/3D reference objects of organs to ASs and CTs, and Organ Mapping Antibody Panels (OMAPs^34^,see below) to CTs and Bs. This definition is adapted from a related publication^2^.

**• HRA Digital Objects (DOs):** HRA DOs are the data components for generating the HRA KG. They are explained in detail in the **Methods** section. ASCT+B tables, 3D reference objects, and OMAPs are examples of HRA DOs that are processed to become part of the HRA KG.

**• Linked Open Data (LOD):** A common data sharing pattern^114^, developed for the Semantic Web (www.w3.org/2001/sw/wiki/Main_Page) that describes how to structure and share semantically rich data that allows for maximum reuse and utility. To be LOD, the data should have an open license, use URIs in the data to name entities whose URIs resolve (i.e., can be queried either directly via web request or via SPARQL) to retrieve structured data in RDF about that URI and link to other resources via URIs.

**• Linked Data Modeling Language (LinkML):** LinkML (linkml.io/linkml)^74^ is a flexible language that allows us to author linked data schemas in YAML (yaml.org) which describe the structure of one’s data. Additionally, it is a framework for working with and validating data in a variety of formats such as JSON, RDF, and tab-separated values (TSV), with generators for compiling LinkML schemas to other frameworks.

**• Organ Mapping Antibody Panels (OMAPs)**: Tabular data structures with panels of experiment-derived and tested antibodies to target proteins for identifying ASs, CTs, cell states, or cell membrane staining in organs. A related publication detailing OMAP construction and usage is available^34,101^.

**• Persistent Uniform Resource Locator (PURL):** A type of URL that points to a resolution service rather than a website. The resolution service then uses content negotiation to determine what content is needed (e.g., HTML for humans, structured data for machines) and redirects the user or directly returns the relevant data. PURLs are used in LOD to provide persistent, resolvable URIs for entities so that they can be referenced without the URIs changing.

**• Resource Description Framework (RDF):** RDF is a standard to represent structured data on the web. It defines relationships between data objects, enabling exchange of structured information through triples consisting of a subject, predicate, and object (www.w3.org/RDF). In the HRA KG, every RDF graph is made available in a series of formats, see **Data Processing Pipeline** in **Methods**.

**• SPARQL Protocol and RDF Query Language:** A query language for RDF graphs (www.w3.org/TR/sparql11-query), SPARQL can be used to write declarative code to retrieve triples that describe two entities and their relationship in an RDF graph.

**• Subject matter experts (SMEs):** Individuals who possess specialized training in areas related to HRA construction, such as anatomists, surgeons, clinicians, and physicians. SMEs may have valuable knowledge about entire organ systems, individual organs, or parts thereof, such as their cellular or molecular make-up, or have expertise in experimental procedures.

## Ontologies and KGs

Linking HRA DOs (see Box 1) to ontologies and, by extension, wider domains of expert knowledge is accomplished by the HRA KG (see **Atlas Coverage** in **Methods**). In the biomedical domain, ontologies are widely used to structure data, which is of high relevance to HRA KG construction. For example, the NCBO BioPortal^12^ provides easy access to 1,168 ontologies; similarly, the European Molecular Biology Laboratory’s European Bioinformatics Institute (EMBL-EBI) Ontology Lookup Service (OLS)^17^ supports 267 ontologies. The Uber-anatomy ontology (Uberon)^18^ is a cross-species ontology representing body parts, organs, and tissues, primarily focused in vertebrates. The Cell Ontology (CL)^19^ is also a cross-species ontology but focuses on classifying and describing cells. Given that Uberon and CL are linked data, one can determine assertions such as *kidney cortical cell* (purl.obolibrary.org/obo/CL_0002681) is *part of* (purl.obolibrary.org/obo/BFO_0000050) *cortex of kidney* (purl.obolibrary.org/obo/UBERON_0001225) from the knowledge represented in these ontologies.

Ontologies are an indispensable part of generating, using, and maintaining KGs as they enable unifying nomenclature across assay types, organs, donors, teams, and consortia. A recent publication by He *et al*.^20^, featuring the HRA, shows how ontologies can be used to model, integrate, and reason over previously siloed clinical, pathological, and molecular kidney data for precision medicine. It highlights the development of the precision medicine metadata ontology (PMMO) to integrate dozens of variables between the Kidney Precision Medicine Project (KPMP)^21,22^ and Chan Zuckerberg Initiative (CZI) CELLxGENE (CxG) data. It then shows specific use cases in detecting healthy vs. acute kidney infection (AKI)/chronic kidney disease (CKD) disease states in cells supported by PMMO, Kidney Tissue Atlas Ontology (KTAO), and the HRA’s CCF Ontology, described in a related publication^23^.

Biomedical KGs use and interlink multiple ontologies to store and query data. For example, the Unified Medical Language System (UMLS)^24^ “metathesaurus” contains approximately 3.4 million biomedical concepts, updated every six months in May and November and is derived from other biomedical terminologies and ontologies. The Petagraph KG^25^ uses the UMLS metathesaurus to integrate biomolecular datasets and connects them to approximately 200 cross-referenced ontologies to support exploration of gene variant epistasis as well as biological assertions with reduced dimensionality, and to link relevant features to chromosome position and chromosomal neighborhoods. The Human BioMolecular Atlas Program (HuBMAP, hubmapconsortium.org)^26,27^ Unified Biomedical Knowledge Graph^2^ (UBKG, ubkg.docs.xconsortia.org and on GitHub^28^) connects HuBMAP experimental data to ontologies. The Scalable Precision Medicine Open Knowledge Engine (SPOKE, spoke.ucsf.edu)^29,30^ processes 41 databases (53 million edges) and 11 ontologies to create an integrated graph with user access via a RESTful API. Petagraph, HuBMAP, and SPOKE use the Neo4J graph platform (neo4j.com). A comparison of the HRA KG and other KGs is provided in the **Technical Validation** section. KGs can be used to extract knowledge across constantly evolving ontologies and data in various states of accessibility (private and public). Efforts like BioCypher (biocypher.org)^31^ enable the rapid construction and maintenance of KGs at lower cost. This also addresses the lack of reusability and integrability, where KGs are built manually for a specific task, and, as a result, do not adhere to Findable, Accessible, Interoperable, and Reusable (FAIR)^32^ principles.

## Updates since the CCF Ontology Paper

The specimen, biological structure, and spatial ontologies in support of the HRA v1.2 using the CCF Ontology v2.0.1 were introduced in a prior publication^23^ in 2023. Given substantial expansions of the HRA data and new user requirements, major CCF ontology changes were made for HRA v2.0, published in December 2023. For example, the CCF Ontology (v3.0) is separated from the HRA, which is represented as a DO of type *collection*. CCF v3.0 is now a DO of type *vocab*, see **HRA Digital Objects** in** Methods**. Before HRA v2.0, the CCF and HRA were a single graph, consisting of the HRA *collection* plus the CCF Ontology embedded into it. Starting with HRA v2.0, the HRA *collection* references the CCF (but does not contain it) and is compiled from a collection of curated HRA DOs; it is hosted by the HRA KG at purl.humanatlas.io/collection/hra/v2.2. In other words, this was implemented to cleanly separate the CCF Ontology from the HRA, thus establishing a boundary between the framework for creating atlases, which is the CCF, and a specific atlas, the HRA.

## HRA vs. HRA KG

The HRA KG makes it possible to access HRA data efficiently and to ask biological questions via programmatic queries. For example, (1) researchers can leverage the KG to identify the number of datasets and B expression levels given a particular CT as identified by its CL ID; (2) computational biologists can identify the canonical Bs for a given CT across many organs through the ASCT+B tables, made available in a validated, processed, enriched LOD format through the HRA KG; (3) and data providers can validate their cell type annotations against the HRA KG or predict the spatial origin of human tissue given its cellular make-up by comparing it against cell type populations in the HRA KG. Examples are provided in the **Usage Notes** and on the companion website at cns-iu.github.io/hra-kg-supporting-information/#basic-usage.

The HRA KG is composed of multiple named graphs (subgraphs), each focusing on a specific part of the atlas, such as biological structures or spatial references. The HRA *collection* is a collection of HRA DOs with DOIs (ASCT+B Tables, 3D reference objects, OMAPs, etc.) that make up the core of the HRA at each release. When processed, it compiles to a (large) RDF graph and is hosted by the HRA KG at purl.humanatlas.io/collection/hra. This relationship is further explained and visualized in the section entitled **The HRA KG in the HRA Ecosystem**.

The HRA uses a graph structure (as opposed to a relational database)^33^ to ensure: **(1) Flexibility**. The schema of the HRA KG can be extended as needed when new organs or HRA DO types become available (as opposed to a rigid table schema that would need to be chosen for a relational database). Many HRA DOs, such as 3D reference objects, are non-tabular and highly nested, which is challenging to model in relational databases.**(2) Support for disparate data**. HRA DOs take on many forms. For example, ASCT+B tables capture ASs, CTs in those ASs, and the Bs that characterize those CTs; they are linked to OMAP and Antibody Validation Report (AVR)^34^ tables (tabular data), 2D images and 3D models (graphic assets), as well as *graph* DOs, such as scientific literature connected to the HRA (HRAlit)^35^ and cell type populations of the HRA (HRApop)^1^, all nested. A relational database would make it necessary to choose a schema for each of these DO types. Moreover, a key strength of KGs lies in their underlying structure: while relational databases rely on tables with relationships stored as foreign keys, graphs are built on nodes and edges; the connections between data points are more obvious and explicit. As a result, unlike relational databases, where linking the data must be inferred through complex joins, graphs are more intuitive and natural for representing data relationships. **(3) Answering biological questions across HRA DO types**. The KG structure makes it possible to programmatically answer questions across multiple DOs for one entire organ via graph queries (e.g., the ASCT+B table for the kidney and the 3D reference object for the female, left kidney). In a relational database, this would necessitate a set of new tables that would need to be carefully created with foreign keys and relationships to support the kind of dynamic graph-based queries readily available in SPARQL. **(4) Deployment as 5-Star LOD**. RDF graphs can be imported into triple stores in their native format and easily be queried together with connected biomedical ontologies (genes, proteins, cells, anatomy), which are also published as RDF. Existing HRA KG queries bridge theaforementioned Uberon^18^ and CL^19^ plus Provisional Cell Ontology (PCL)^36^, HUGO Gene Nomenclature Committee (HGNC, www.genenames.org)^37^, and HRA nodes, properties, and relationships stored or imported from their respective graphs.

To use the HRA KG in connection with a relational database, an initial query into the HRA KG can be used to retrieve data as a simple table; then, a database management system like PostgreSQL (www.postgresql.org) can be used to aggregate the data with SQL features that perform complex window functions on it. This way, data from the HRA KG can be indexed and used in a database downstream.

## Limitations

The current HRA KG has a number of known limitations that will be addressed in future HRA releases:

### Automation

While many parts of the HRA KG construction process are automated, collecting and providing DOs in their original form, such as comma-separated values (CSV), binary Graphics Library Transmission Format (glTF/GLB, www.khronos.org/gltf), or scalable vector graphics (SVG), is still a manual process involving human labor. In future releases, machine learning algorithms^15,38–45^ will be employed to speed up tissue data segmentation and annotation, using human expertise to review and correct as needed but not manually compile DOs.

### Build Time

At present, building the HRA KG from unprocessed DOs using code on GitHub^46^ takes about 13 hours on a local Linux server with 256GB RAM and 20 cores. As new DO types are added and HRApop as well as HRAlit grow, the construction process will be continuously optimized by implementing better KG structures, using parallelization, and improving normalization and enrichment code (some libraries are particularly slow for certain DO types). Preliminary results from one experiment showed a nearly one-third reduction in execution time, demonstrating the potential of parallelization in faster, more efficient KG construction.

### Reduce *ASCTB-TEMP* Terms

As of HRA v2.2, 221 CTs across 33 ASCT+B tables do not yet exist in CL or PCL; instead, they have an *ASCTB-TEMP* expert provided label. GitHub issues have been submitted for all, and the EMBL-EBI team is adding these terms. The **Technical Validation** section details ongoing efforts to add new AS and CT terms from the HRA KG to existing ontologies. As of May 2025, 162 AS terms were added to the Uberon^18^ ontology, 155 CTs were added to CL^19^, and 468 CTs were added to PCL^36^ including 461 for the human brain^47^ in support of HRA construction and usage.

### Data Modeling

To be most useful to the HRA KG, each new DO type must have a LinkML schema (see Box 1), normalization code, and enrichment code to transform the raw data into queryable information. As new use cases are identified, the HRA structure and canned queries will be revised and expanded. Currently, HRAlit^35^ is being served via a relational database. Knowledge modeling is underway to create an HRAlit graph and to properly connect it to the HRA KG, which will allow users to query peer-reviewed literature and funded awards for entities in the HRA KG.

### Ease of Use

Retrieving data from KGs requires experience writing SPARQL queries, which few clinicians and biomedical researchers possess. The HRA KG comes with canned queries at apps.humanatlas.io/api/grlc/ as well as Jupyter Notebooks (see companion website at cns-iu.github.io/hra-kg-supporting-information). Going forward, the HRA Documentation Portal (under construction) will help train and provide resources to users learning how to query the HRA KG. Additionally, since KGs offer access to structured data, the possibilities of utilizing large language models (LLMs) are explored to allow users to ask questions in prose. An LLM, enhanced by retrieval-augmented generation (RAG), was prototyped to support natural language queries that are informed by the knowledge in the HRA KG, see companion website at cns-iu.github.io/hra-kg-supporting-information/#using-llms-and-rag-with-hra-kg. Finally, the HRA KG Explorer UI is in development, which will allow users to browse the KG via the web to quickly identify, select, and download HRA DOs of interest in all available graph formats. This will enable easy access to the HRA KG to users without experience writing code, making API requests via the grlc.io service, or running SPARQL queries.

## Methods

### HRA Digital Objects

HRA DOs come in diverse formats, such as ASCT+B tables^1^ (humanatlas.io/asctb-tables), 3D reference objects (humanatlas.io/3d-reference-library, 3d.nih.gov/collections/hra), and OMAPs^34^ (humanatlas.io/omap), see all in Box 1. Each DO has a type, name, and version in a PURL (see Box 1). For example, the PURL for the ASCT+B table for the kidney is purl.humanatlas.io/asct-b/kidney/v1.6, where *asct-b/kidney/v1.6* indicates *asct-b* as the type, *kidney* as the organ name, and *v1.6* as the version. These DOs are provided by SMEs (see Box 1) using well-defined data structures (see SOPs at humanatlas.io/standard-operating-procedures for details), and are regularly validated^48^.

HRA DOs allow SMEs to provide expert knowledge that can be queried programmatically. ASCT+B tables, for example, make it possible for anatomists, surgeons, and other experts to digitize knowledge about the CTs and Bs in healthy tissue via Google Sheets. When constructing a table, SMEs are asked to crosswalk HRA terms for AS, CT, and B to terms and IDs in existing ontologies. Parsing the Google Sheets for downstream HRA usage is not advisable, as detailed validation and additional enrichment are required before the ASCT+B table data can be used.

**Fig. 1:**
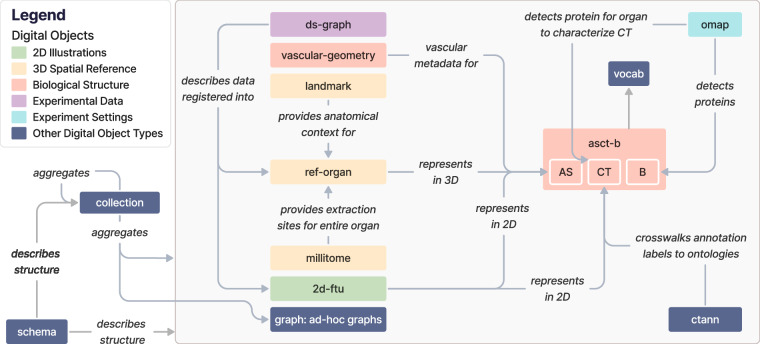
The 13 DO types in the HRA KG and how they relate to each other. Note that underscores in edge labels were replaced with blank spaces for legibility. Entity-relationship (ER) diagrams are provided on the companion website at cns-iu.github.io/hra-kg-supporting-information/#mermaid-diagrams.

A complete list of the 13 DO types in HRA v2.2 is provided in Table 1. These DOs can be categorized as reference data (*2d-ftu, asct-b, ctann, landmark, millitome, omap, ref-organ, vascular-geometry*), experiment data (*ds-graph, graph*), and other data (*collection, schema, vocab*). DOs are available in a variety of formats on the LOD server at lod.humanatlas.io. Fig. 1 illustrates high-level relationships among the 13 DO types.

**Table 1:** Different DO types used in the HRA KG, describing their purposes and the data they contain plus SOPs detailing their construction.

DO	Description
**Reference Data**	
2d-ftu	Provides 2D illustrations of FTU structures in an organ, with image assets and cell annotations that assign proper labels and identifiers based on CL for each image segment. An SOP is available^98^.
asct-b	Represents an ASCT+B table, see Box 1. Contains detailed knowledge about human anatomy in a nested, hierarchical order, explaining the organization of ASs, the CTs in each AS, and the Bs that distinguish each CT^1^. An SOP is available^99^.
ctann	Represents a crosswalk, see Box 1. Translates CT labels or abbreviations from sc/snRNAseq analysis tools, specifically Azimuth^49^, CellTypist^50,51^, and popV^52^ into standardized terms in CL and PCL. The translation quality is measured using standard predicates in SKOS, such as* skos:exactMatch* and *skos:narrowMatch narrow match*, to ensure consistent data harmonization across sc/snRNAseq analyses.
landmark	Provides 3D model shapes representing features near organs of interest (e.g., an artery or pelvis bone near a kidney) to help experts accurately orient themselves when registering tissue blocks into a 3D reference object.
millitome	Provides data about cutting tissue samples using a millitome device. An SOP is available^100^.
omap	Represents an OMAP, see Box 1. Reduces the costs of conducting cell imaging experiments. OMAPs^34^ contain a panel of antibodies designed to target specific proteins for identifying ASs, CTs, cell states, or cell membrane stainings within organs, based on actual experimental projects. An SOP is available^101^.
ref-organ	Represents a 3D reference object, see Box 1. Provides 3D models of human organs with accurate size and position data, to support the creation of a comprehensive 3D model of the human body. Each 3D model is carefully annotated with a proper label and an identifier from Uberon or FMA. Multiple SOPs are available^102,103^.
vascular-geometry	Provides detailed geometry information on the human blood vasculature with key attributes, such as diameter, length, population, sample size, and reference to the source of data. Multiple SOPs are available^104–107^.
**Experiment Data**	
ds-graph	Provides sample registration information submitted by consortium members in HuBMAP and other efforts, including accurate position, rotation, and size. When combined with *ref-organ* data, this information helps create 3D tissue block placements. This tissue block information is linked to datasets from researchers’ assay analyses that offer deeper insights into the tissue blocks. The “ds” stands for “dataset.”
graph	Contains externally created RDF (see Box 1) graph data, i.e., produced by a process different from the hra-do-processor (see **HRA KG Construction and Deployment** in **Methods**).
**Other Data**	
collection	Combines multiple DOs to create a collection of data. The HRA itself is released as a curated *collection* of DOs in each new HRA KG version.
schema	Describes the structure of the normalized form of a single DO type, its metadata, or shared concepts between DOs.
vocab	Contains various reference ontologies and vocabularies that hold standard concepts and relationships used to construct DOs. *vocab* DOs are typically external biomedical ontologies like CL and Uberon; they provide a convenient mechanism for querying reference ontologies alongside HRA-curated DOs.

### High-level Relationships between DO Types

2D Illustrations (green): *2d-ftu* DOs represent ASs in 2D (because FTUs are ASs) and the CTs in them, based on experimental data. *2d-ftu* DOs can be downloaded in their processed form or as SVG, Portable Network Graphics (PNG), or Adobe Illustrator (AI) files. The crosswalks from 2D FTU illustrations to ASCT+B tables are also available as* 2d-ftu* DOs and can be downloaded as CSV files.

3D Spatial Reference (yellow): *ref-organ* DOs get anatomical context from *landmark* DOs, and *millitome* DOs provide extraction sites for an entire organ, represented by a *ref-organ* DO. *ref-organ* and *landmark* DOs can have arbitrary 3D shapes and are available as GLB files. *millitome* DOs are cuboids and can be downloaded in JSON-LD. The crosswalks from 3D reference objects to ASCT+B tables are also available as* ref-organ* DOs and can be downloaded as CSV files.

Biological Structure (pink): The *asct-b* DO type plays a central role for multiple other DO types, see Box 1 and Table 1. *vascular-geometry* DOs provide vascular metadata for *asct-b* DOs. Both can be downloaded as CSV files.

Experimental Data (purple): *ds-graph* DOs describe experimental datasets mapped into a *ref-organ* and enriched with further metadata, such as cell type summaries and mesh collisions. They can be downloaded as JSON-LD files.

Experiment Settings (cyan): *omap* DOs enable detection of proteins and CTs and are thus connected to ASs and Bs in *asct-b* DOs. *omap* DOs are specific to organs, tissue preservation methods, and assay types and can be downloaded as CSV files or Microsoft Excel spreadsheets (XLSX).

*Other DO types (blue): ctann* DOs represent CTann crosswalks (see Box 1) that map manual and machine learning annotations for CTs from different CTann tools such as Azimuth^49^, CellTypist^50,51^, and popV^52^ to the ASCT+B tables via ontologies such as Uberon^18^, FMA^53,54^, CL^19^, and PCL^36^. Like the aforementioned *omap* DOs, *ctann* DOs allow mapping experimental datasets, represented as the aforementioned *ds-graphs*, into the HRA. Both can also be downloaded as CSV and XLSX files. *vocab* DOs are referenced by *asct-b*, *omap*, *ctann*, and *ref-organ* DOs to annotate ASs, CTs, and Bs with ontology terms and can be downloaded as Web Ontology Language files (OWL, www.w3.org/OWL). *graph* DOs are ad-hoc graphs that can reference any other DO type as needed, depending on their function and scope, and can thus have any download format available for the referenced DOs. All current *graph* DOs in the HRA KG are listed in Table S1. *collection* DOs aggregate multiple DOs and can be downloaded as YAML files, allowing end users to create customized configurations for their particular needs. Importantly, the HRA itself is a *collection* DO, with the most recent release always available at lod.humanatlas.io/collection/hra/latest. All current* collections* in the HRA KG are listed in Table S2. Finally, the *schema* DO type describes the structure of all HRA DO types plus their metadata and can be downloaded in a variety of formats, including YAML, PNG, and SVG (as an ER diagram).

### Metagraph

**Fig. 2:**
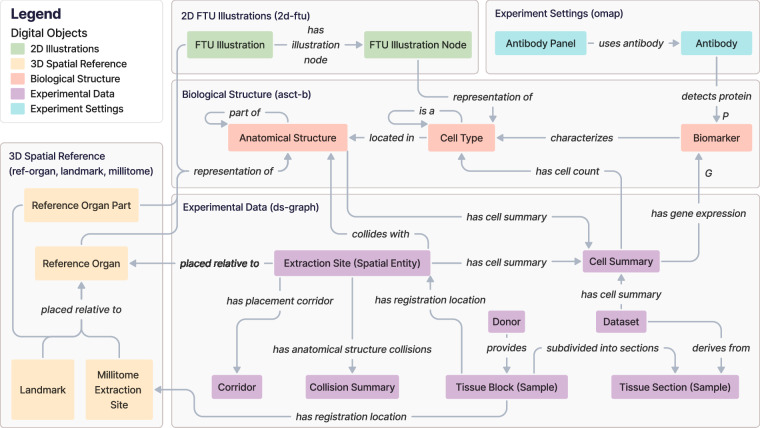
The HRA KG metagraph illustrates high-level relationships between subgraphs. Note that all edge labels have been modified as needed to avoid overlap while maintaining correct semantics. For class names (e.g., *FTU Illustration*), a blank space was added between PascalCased class names.

HRA DO types can be aggregated into five thematic subgraphs: 2D Illustrations, 3D Spatial Reference, Biological Structure, Experimental Data, and Experiment Settings. The HRA KG metagraph in Fig. 2 depicts the higher-order relationships among these interconnected subgraphs. The 3D Spatial Reference subgraph (yellow) specifically is presented and explored in detail in a prior publication on the CCF Ontology^23^.

The **2D Illustrations** subgraph uses the *Biological Structure* subgraph to enrich its 2D *FTU Illustrations* and the *FTU Illustration Nodes* inside of it with the ontology-aligned naming for FTUs and CTs. The *Biological Structure* subgraph provides the 2D anatomical context for the ASs and CTs in the FTUs.

The **3D Spatial Reference** subgraph represents the 3D CCF used to accurately position 3D reference objects for organs, anatomical landmarks inside or adjacent to them, and millitomes (see Table 1) within the human body. This subgraph links to the *Biological Structure* to provide the 3D anatomical context for the ASs.

The **Biological Structure** subgraph anchors all other components. It contains the ASs, CTs, and Bs from 33 ASCT+B tables together with their ontological relationships (for 32 organs plus one for anatomical systems). ASs and CTs can have self-loops, where an AS can be *part_of* another AS, creating a partonomy, and a CT can be a subclass of another CT in a typology (*is_a*).

The **Experimental Data** subgraph focuses on experimental *Datasets* generated from assay analyses performed on *Donor Tissue Blocks (Samples)*. These are assigned an *Extraction Site (Spatial Entity)* with the HRA Registration User Interface (RUI)^55^ based on their anatomical origins to provide a location within the CCF. Since HRA v1.2, extraction sites are *placed_relative_to* *Reference Organs*; note that this is also a change in terminology, which used to be called *has_placement*, see prior publication^23^. All possible alternative locations of an *Extraction Site (Spatial Entity)*, given its intersection(s) with one or multiple 3D ASs, are captured in a *Corridor*. Systematic whole-organ registration is supported by the *Millitome*, which defines a set of connected extraction sites *placed_relative_to* a *Reference Organ*. This subgraph also accommodates derived data computed from the assay results and extraction sites, such as (a)* Cell Summaries*, which provide cell type populations and mean gene expression values for specific CTs and their associated datasets and 3D extraction sites, and (b) *Collision Summaries*, which identify ASs that overlap with the registered tissue blocks inside a 3D extraction site, and detail the precise intersection volume and percentages in these collisions.

External annotations that are not shown in this subgraph are also possible: *Datasets* can be annotated with a publication; *Donors*, *Tissue Blocks (Samples)*, and *Datasets* can have separate links to a data portal; and *Donors* can be annotated with a tissue provider.

Finally, the **Experimental Settings** subgraph catalogs *Antibody Panels* via OMAPs^34^, capturing details of specific *Antibodies* used to detect particular protein* Biomarkers* in the *Biological Structure* subgraph.

### The HRA KG in the HRA Ecosystem

The HRA KG represents major DOs of the HRA v2.2, including 33 ASCT+B tables, 23 OMAPs, 22 2D FTUs, 71 3D reference objects (plus two whole body models with all organs for male/female and a crosswalk from 3D ASs to ontology terms), see apps.humanatlas.io/dashboard/data. In May 2025, the HRA KG had 10,064,033 nodes, 171,250,177 edges, and a size of 125.84 GB. The size of the 71 3D reference objects (GLB files, lod.humanatlas.io/ref-organ) in HRA v2.2 is 301 MB. In addition, the data covers anatomical landmarks which are used in the RUI to facilitate tissue block registration in 3D reference objects; these are available at lod.humanatlas.io/landmark. As of HRA v2.2, there are landmarks for 59 out of 71 3D reference objects. Together, they are 261 MBs large.

**Fig. 3:**
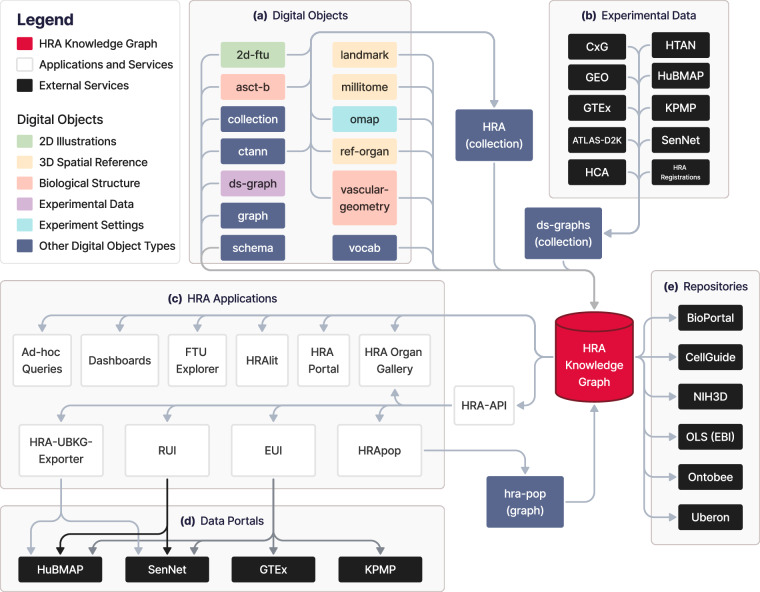
The role of the HRA KG in the HRA data ecosystem: **(a)** HRA DOs, **(b)** experimental datasets as well as 3D size, location, and rotation of tissue blocks registered into the HRA, **(c)** HRA applications, **(d)** data portals, and **(e)** external services of the HRA KG. Note that the HRA KG is able to serve all existing versions of the HRA and HRA DOs, including those preceding the most recent HRA v2.2 release in December 2024.

To make raw and processed HRA DOs programmatically available as RDF graphs, the HRA KG serves as the primary database for the HRA, see Fig. 3.

**(a)** 703 individual DOs of 13 types go through a 3-step process of normalization, enrichment, and deployment via the **hra-do-processor** (see code on GitHub^56^), where they are transformed from raw data in miscellaneous file formats into an RDF graph, resulting in the HRA KG. Together, all the DOI’d HRA DOs form the HRA *collection*. The normalization, enrichment, and deployment processes are described in the **HRA KG Construction and Deployment** section below.

**(b)** Graph representations of external experimental datasets from various sources with mean B expression values and cell type population data, resulting in the *ds-graph* DO type. Experimental data from various portals is mapped into the HRA via one or a combination of multiple methods, such as 3D tissue registration with the RUI^55^, Azimuth^49^, CellTypist^50,51^, and popV^52^ annotations aligned to ASCT+B tables via ontology crosswalks, or OMAPs^34^ for spatial proteomic data. These portals include the CZI CxG portal (cellxgene.cziscience.com), the Gene Expression Omnibus (GEO, www.ncbi.nlm.nih.gov/geo)^57,58^, the Genotype-Tissue Expression Portal (GTEx, gtexportal.org/home)^59^, the Analysis, Technology, Leadership, Administration, and Science - Data to Knowledge (ATLAS-D2K) Center, which contains data for the GenitoUrinary Developmental Molecular Anatomy Project (GUDMAP, www.atlas-d2k.org/gudmap)^60,61^ and the (Re)Building a Kidney (RBK, https://www.atlas-d2k.org/rebuildingakidney) Consortium^62^, the Human Cell Atlas (HCA, www.humancellatlas.org/data-portal)^63^, the Human Tumor Atlas Network (HTAN, humantumoratlas.org/explore)^64^, the HuBMAP^26,27^ Data Portal (portal.hubmapconsortium.org), the KPMP^21,22^ Kidney Tissue Atlas (atlas.kpmp.org), and the Cellular Senescence Network (SenNet, data.sennetconsortium.org/search)^65^ Data Portal. Finally, hra-registrations refers to HRA DOs of type *ds-graph* that are manually curated, stored in a HRA GitHub repository, and used in the EUI and other HRA applications. The HRA API provides dedicated endpoints to retrieve the latest *ds-graph* DO data from various portals, e.g., apps.humanatlas.io/api/ds-graph/hubmap for HuBMAP. 

**(c)** HRA applications and services use the HRA KG as their main data backend through the HRA API or via a SPARQL endpoint (lod.humanatlas.io/sparql).

The HRA API provides the HRA UIs with access to Uberon, FMA, CL, PCL, and HGNC IDs for ASs, CTs, and Bs as well as spatial entities for tissue blocks and organs^23^ for the RUI and Exploration User Interface (EUI)^55^. For example, when using the EUI to select AS, CT, and B terms (on the left side of the UI), counts are retrieved from those relationships that are curated from multiple graphs. Since all graphs are in the RDF format, multiple graphs can be queried seamlessly without modifying the source graphs. The primary HRA API at apps.humanatlas.io/api has programming language-specific client libraries in JavaScript, TypeScript, Angular 17+, and Python 3.6+. These client libraries are published to common code package managers, including NPM (www.npmjs.com) and PyPI (pypi.org), that wrap API calls into simple function calls, making HRA data easy to use from software development environments. A full list of client libraries is available at humanatlas.io/api. A set of example Python Notebooks is provided on GitHub^66^. Links to publicly accessible instances of UIs using the HRA KG on data portals are provided in Table S3. Ad-hoc queries to retrieve counts and access DOs from the HRA KG can be run via the SPARQL endpoint. The HRA Dashboard (apps.humanatlas.io/dashboard) and the HRA Portal (humanatlas.io)^2^ provide usage and data statistics about the HRA by querying the HRA KG. The FTU Explorer (apps.humanatlas.io/ftu-explorer)^67^ accesses CL IDs for cells and HGNC IDs for Bs via the HRA KG. HRAlit data^35^ (see also GitHub^68^) connects 136 DOs from HRA v1.4 to 583,117 experts, 7,103,180 publications, 896,680 funded projects, and 1,816 experimental datasets. HRApop^1^ provides CTs and mean B expression values for experimental datasets mapped to the HRA. The HRA Organ Gallery in virtual reality (VR)^69^ utilizes the HRA KG to show predicted CTs in tissue blocks in immersive, 3D space.

**(d)** The HRA KG is used in several **data portals**, including some from which *ds-graph* DOs are being extracted (HuBMAP, SenNet, GTEx, KPMP). For example, the HRA-UBKG Exporter^70^ is used to make HRA data available for HuBMAP Data Portal services (portal.hubmapconsortium.org), such as Uberon^18^ aligned organ pages (e.g., portal.hubmapconsortium.org/organ/lung), and AS search and filter functionality. The RUI and EUI are used in various portals to serve consortium-specific needs of tissue providers.

**(e)** Several **external repositories** serve and/or use HRA KG data: NCBO Bioportal and Ontobee host the HRA CCF Ontology^23,71,72^ (see Box 1). The OLS by EMBL-EBI provides a collection of HRA DOs for validation^73^. CxG CellGuide (cellxgene.cziscience.com/cellguide) utilizes the ASCT+B tables to identify and present canonical Bs for CTs to their users. Finally, the NIH3D platform by the National Institute of Allergy and Infectious Diseases (NIAID) hosts all 71 3D reference objects for organs in the HRA v2.2 (3d.nih.gov/collections/hra), plus two United files (one male, one female) with all respective organs combined. Additionally, the NIH BioArt platform serves 22 2D FTU illustrations at bioart.niaid.nih.gov/discover?collection=2.

### HRA KG Construction and Deployment

All data and code to construct, deploy, and use the HRA KG is available on GitHub^46^, Zenodo, or via APIs, see details in Table S4. The HRA KG is constructed twice a year, synchronized with the HRA release cycle^2^ (see release notes at humanatlas.io/overview-training-outreach#release-notes). The most essential code piece, the hra-do-processor^56^, is built around three main components: the **schema**, the **data processing pipeline**, and the **web infrastructure**. The following sections detail each of these components.

### Schema

Well-defined data schemas are crucial for ensuring data consistency, interoperability, and validation in data management and analysis. The HRA KG uses LinkML^74^, a flexible and user-friendly schema language designed to create effective data models and validation tools, to ensure input data adheres to a defined schema. ER diagrams of core HRA schemas explain the relationships between different HRA DOs, see examples on the companion website at cns-iu.github.io/hra-kg-supporting-information/#mermaid-diagrams. A complete listing of the LinkML schemas used in HRA KG construction is available in Table S5. In addition to structural constraints, LinkML supports the implementation of reference integrity to ensure that linked entities conform to external ontologies. For instance, the *binds_to* slot in the *Antibody* class was explicitly constrained to accept only HGNC codes, preventing invalid associations with non-protein entities. Furthermore, LinkML enables explicit mappings between classes or slots to standardized vocabularies and ontologies. For example, the same *binds_to* slot was mapped to the *binds_to* property in the CCF vocabulary.

### Data Processing Pipeline

The 13 HRA DO types described above come from SMEs who contribute their knowledge of anatomy, antibodies, pathology, and experimental procedures, and from public data portals as well as repositories. Manually curated and experimental datasets from diverse sources need to be mapped to the HRA and standard ontologies, normalized to a standard format (e.g., unification of term labels), and enriched (e.g., linked to existing ontologies in support of causal reasoning). The hra-do-processor normalizes and enriches these DOs, then deploys them as RDF graphs. A catalog of these graphs is available on the HRA KG LOD server at lod.humanatlas.io. A sequence of five steps converts raw DOs into the HRA KG. The steps are normalization, enrichment, deployment, finalization, and serving, see details below.

#### Normalization

This initial step ensures that all incoming data is transformed into a consistent format that aligns with the predefined schema. The hra-do-processor loads and parses the disparate source data and transforms it into a standardized linked data representation. For example, in the case of *asct-b* DOs, the source data comes from Google Sheets, exported as CSV files (unnormalized, raw ASCT+B tables are available at humanatlas.io/asctb-tables); during normalization, the hra-do-processor reads this tabular structure and converts each data row in the table into a tree structure, see example shown in Fig. S1.

An exemplary YAML file is provided at github.com/cns-iu/hra-kg-supporting-information/blob/main/docs/intermediary_format.yaml. YAML was chosen as the standard file format for the normalized data due to its simplicity, readability, and interoperability with JSON, as well as its high-level of support in LinkML. By converting the LinkML schema into a JSON schema file, the translated YAML data can easily be validated to ensure it adheres to the defined schema and the automated ingestion code is correctly implemented.

In general, HRA DOs come in two major formats: tabular data (like *asct-b* DOs) and nested key-value pairs (like *ds-graph* DOs). SOPs provide detailed instructions to SMEs on how to author DOs, e.g.,* asct-b* and *omap* (see references to relevant SOPs in Table 1). Extracting knowledge from tabular data begins with schema inference, which includes identifying header rows, data types, and relationships between columns. Typically, the first row serves as the header, and the data types are inferred by analyzing the values in the entire column. Determining the relationships between columns requires either consulting a knowledge base or relying on domain experts^48^. In *asct-b* DOs, the relationship between the column headers for ASs at different levels (*AS/1*, *AS/2*, *AS/3*) involves aligning their values with Uberon or FMA, then confirming that they follow the *part_of* relationship. Further refinement means identifying parent-child relationships by grouping columns that logically belong together as a single concept. For example, the columns *rrid*, *host*, *isotype*, *clonality*, and *conjugate* in an *omap* DO are attributes of an antibody rather than separate concepts. Once the table structure is understood, the next step is semantic annotation, where table components are linked to concepts from external knowledge sources or ontologies. This process involves three steps: (1) matching individual cell values to ontology terms, such as linking the label *kidney* to *UBERON:0002113*; (2) associating entire columns with controlled vocabularies, for example, defining the *REF/*1 column as *dct:references* to describe a cited, related resource, and (3) associating relationships between columns to ontology terms, such as linking the relation between *AS/2* and *AS/1* columns to *BFO:0000050*, which denotes a *part_of* relationship.

For nested key-value pair data, schema inference is more straightforward since the hierarchical structure is more visible than in flat, tabular data. However, it is still necessary to determine data types, structural relationships, and references within the data. For instance, the *size* field in data from *ref-organ* DOs is recognized as a nested object that contains *x*, *y*, and *z* fields, which are later inferred to be of decimal type based on the populated values. When identifying references, fields containing the substring *id* are typically recognized as reference fields that indicate a link to an external entity.

Once the schema is inferred, a similar step of semantic annotation is performed to the data elements by linking them to ontology concepts. For example, a nested field *node.size.x* is mapped to the *x_dimension* annotation property in the CCF Ontology. Likewise, the value *VH_M_aortic_valve* is mapped to *UBERON:0002137*, allowing it to be correctly identified as an aortic valve from Uberon, complete with its anatomical context. Finally, references are introduced using unique identifiers to maintain consistency and avoid redundancy. For instance, Research Resource Identifiers (RRIDs)^75,76^ are used to refer to a specific antibody rather than repeating its full details.

Data quality is crucial to ensure accurate query results. To handle potential errors in the source data, e.g., incorrect or ambiguous entity names, the normalization step (1) performs basic string validation, e.g., ensuring that DOIs begin with the right prefix, (2) ensures that Compact URIs (CURIEs) are formatted correctly, (3) applies custom validation per HRA DO type (optional), and (4) validates the structure of the normalized data against the inferred schema.

#### Enrichment

This step converts the normalized data for all DOs to RDF and enriches it with relationships, entities, and metadata from external resources. After the source data is translated into YAML and validated, enrichment begins by converting the validated data into OWL-based statements. The LinkML framework offers tools that facilitate the transformation of tree-structured data into OWL constructs, including class and property declarations, as well as instances of a class (see Fig. S2). OWL was chosen as the data representation for the enrichment step due to its robust capabilities for rich data expression, its ability to embed semantic meaning, and its seamless integration with LinkML. In turn, LinkML provides direct support for OWL by allowing schema elements to include OWL constructs, making it easy to map the data into a semantically rich ontology structure. The enrichment process continues by integrating additional information from reference ontologies as well as authoritative databases like RRID and the Antibody Registry^77^ API to retrieve metadata (label, description) about antibodies, for which there is only an RRID in the raw HRA DO for OMAPs.

The goal is to enhance the initial data gathered from SMEs with more detailed, authoritative information. In *asct-b* DOs, many data points already reference Uberon and CL terms. These terms are enriched by retrieving supplementary information from the corresponding ontologies, including class hierarchies, labels, definitions, synonyms, database references, and visual depictions. For example, the standard label for a *CL:0002306* is identified as *epithelial cell of proximal tubule*, which is categorized under the broader class *meso-epithelial cell*. These details, which were absent from the original dataset, add valuable context. The end result is a semantically enriched dataset that not only preserves the original data but also extends it with additional context, relationships, and meaning.

#### Deployment

Once the data is enriched, it is prepared for use in downstream applications or for access by end users. This stage involves organizing the data into its final distribution formats and setting up the correct structure for the file system directory. HRA data is used by many different tools that need diverse formats: HRA UIs like the EUI and RUI^55^ use JSON-LD, which is best when using the data directly and imperatively (i.e., in a programming language using *for* loops). Python and JavaScript have native support for handling JSON and have semantics built in, and an exemplary Jupyter Notebook (jupyter.org) to showcase how an ASCT+B table as a JSON file can be parsed is provided on the companion website at cns-iu.github.io/hra-kg-supporting-information/#notebook-to-query-the-hra-knowledge-graph-kg; the **Usage Notes** section provides details. The Blazegraph (blazegraph.com) SPARQL server uses Terse RDF Triple Language (Turtle)^78^; the Turtle format also helps developers write SPARQL queries to the HRA KG by making its triple structure explicit and showing possible subjects, predicates, and objects. Older semantic web tools use RDF/Extensible Markup Language (XML, www.w3.org/TR/rdf-syntax-grammar), N-Triples^79^, and N-Quads^80^. Additionally, ROBOT^14^, Apache Jena, and RDF I/O technology (RIOT, jena.apache.org/documentation/io) use XML for reifying graphs. HRA KG data is preprocessed in those formats to be readily usable. Publishing all these formats streamlines the content negotiation process later (see Box 1), when different applications access the published HRA KG on the LOD server at lod.humanatlas.io, which can then immediately deliver the HRA data in the correct format. During the deployment step, the hra-do-processor also prepares the metadata that accompanies the graph data before copying files and data assets into their designated folders.

#### Finalization

Next, the necessary metadata and HTML landing pages for web publication are generated, e.g., lod.humanatlas.io/asct-b/eye/latest leads to the most recently published ASCT+B table for the eye. In addition, this stage includes building the SPARQL database that will be uploaded to the web for users to access at lod.humanatlas.io/sparql. During deployment, data and metadata for each DO are converted and exported; during finalization,  an indexed and optimized database file for the Blazegraph SPARQL server is also derived. The database contains the latest version of every DO, every version of the HRA *collection*, and a metadata catalog for every version of every DO in the HRA KG.

#### Serving

Data processed in the previous steps, including raw DO data, processed data products, HTML pages, metadata, and the SPARQL database, are made available online at lod.humanatlas.io. The data is regularly updated and synchronized, either during scheduled releases or when updates occur, to ensure that the most current version is always available. To make the processed data widely accessible, Amazon Web Services (AWS, aws.amazon.com) is used to serve the HRA KG as LOD, employing three of its core services: Amazon Simple Storage Service (S3), Elastic Container Service (ECS) and CloudFront for data storage, computation, and content delivery, respectively. Implementation details are provided in the next section.

### Web Infrastructure

*Amazon S3* is a highly scalable data storage service to store and retrieve data. The HRA KG uses it to store the content from the local deployment directories, including the Blazegraph database file. By syncing these local directories with an S3 storage, the data is securely stored and readily available for content delivery.

*Amazon ECS* is a fully managed container service to run applications in Docker containers (www.docker.com) for a highly scalable and reliable environment for computational needs in support of HRA HG construction and usage. For HRA, a Blazegraph instance is run within an ECS container. The ECS container periodically checks the S3 storage for an updated Blazegraph database file. When a newly built Blazegraph file is detected, ECS will seamlessly update the Blazegraph server to ensure that the latest data is available for querying.

*Amazon CloudFront* is a global Content Delivery Network (CDN) designed to accelerate the distribution of content by caching copies at multiple serving locations around the world. The HRA KG uses CloudFront to create a URL fabric that caches and serves content from S3 storage, ensuring fast and reliable access for users regardless of their geographical location. The content stored in S3 is made publicly available through URLs leading to a CDN, e.g., cdn.humanatlas.io/digital-objects/ref-organ/liver-female/v1.2/assets/3d-vh-f-liver.glb, which returns the GLB file for the 3D reference object for the female liver. This allows downstream users of the HRA KG to access processed, high-quality HRA DOs via common data exchange formats, e.g., the Harmonizone^81,82^ procures CTs and genes from the *asct-b* DOs via the HRA CDN at cdn.humanatlas.io/hra-asctb-json-releases/hra-asctb-all.v2.2.json.

Additionally, CloudFront provides advanced content negotiation features, e.g.,  dynamic handling of URLs starting with purl.humanatlas.io and lod.humanatlas.io. Content negotiation allows the web infrastructure to serve data in different formats based on user needs, whether a user requires RDF, JSON, XML, or another format. The PURL returns HRA DO *data* as an RDF graph based on the *Accept* header of the request: human users get redirected to the LOD server, machines to JSON or RDF versions. The LOD server also returns metadata for processed HRA DOs, such as who created it, when it was published, and what different assets and reifications are available to download, as Data Catalog Vocabulary (DCAT) datasets with provenance (www.w3.org/TR/vocab-dcat-3). Moreover, CloudFront also acts as an intermediary for the SPARQL endpoint hosted by Blazegraph within ECS by making it accessible at lod.humanatlas.io/sparql.

### Other Ontologies

The HRA KG includes other reference ontologies at lod.humanatlas.io/vocab (e.g., Uberon and CL) so they can be queried together in an efficient manner. Table 2 lists all ontologies that are included in the HRA KG together with their version numbers.

**Table 2:** Ontologies used in the HRA KG as of HRA v2.2.

Name	Description	Version Number	URL	Main Website
CCF	Common Coordinate Framework Ontology^23^	3.0	purl.humanatlas.io/vocab/ccf	humanatlas.io/ccf-ontology
CL	Cell Ontology^19^	2024-09-26	purl.humanatlas.io/vocab/cl^108^	obophenotype.github.io/cell-ontology
FMA	Foundational Model of Anatomy^53,54^	5.0.0	purl.humanatlas.io/vocab/fma	si.washington.edu/projects/fma
HGNC	HUGO Gene Nomenclature Committee^37^	2024-03-04	purl.humanatlas.io/vocab/hgnc	www.genenames.org
HRAVS	HuBMAP Research Attributes Value Set	2.5.3	purl.humanatlas.io/vocab/hravs	bioportal.bioontology.org/ontologies/HRAVS
LMHA	Cell Ontology for Human Lung Maturation (LungMAP Human Anatomy)^109^	1.4	purl.humanatlas.io/vocab/lmha	bioportal.bioontology.org/ontologies/LUNGMAP_H_CELL
PCL	Provisional Cell Ontology^36,110^	2024-07-11	purl.humanatlas.io/vocab/pcl	GitHub^108^
RO	OBO Relation Ontology (zenodo.org/records/14976337)	2024-04-24	purl.humanatlas.io/vocab/ro	GitHub^111^
Uberon	Uberon Multi-species Anatomy Ontology^18^	2024-11-25	purl.humanatlas.io/vocab/uberon	obophenotype.github.io/uberon
VCCF	Vasculature Common Coordinate Framework^1,112^	2024-02-23	purl.humanatlas.io/vocab/vccf	GitHub^113^

## Data Records

The whole, compressed HRA KG is available on Zenodo^83^ and is ~5.1 GB large. It is also deposited on the HRA CDN at cdn.humanatlas.io/hra-kg-releases/hra-kg.v2.2.tar.xz. The primary server for the HRA KG v2.2 is at lod.humanatlas.io. The SPARQL endpoint to query the HRA KG is at lod.humanatlas.io/sparql. The HRA API (apps.humanatlas.io/api) supports programmatic access to the HRA KG. Exemplary queries are available via the companion website at cns-iu.github.io/hra-kg-supporting-information/#example-queries.

The NCBO BioPortal^84^ hosts both the HRA (mirror of purl.humanatlas.io/collection/hra/v2.2) and the CCF Ontology^23,71^ (mirror of lod.humanatlas.io/vocab/ccf).

The EMBL-EBI OLS hosts the latest versions of both the HRA^73^ and CCF^85^. OLS provides both a web-based GUI for users and programmatic access via the REST API (www.ebi.ac.uk/ols4/help), enabling the HRA and CCF to be accessed using the same standard interface as other ontologies.

The NIH3D platform by NIAID hosts all 71 3D reference objects for organs in the HRA v2.2 alongside two United files with all organs combined (3d.nih.gov/collections/hra). BioArt makes 22 2D FTU illustrations available at bioart.niaid.nih.gov/discover?collection=2.

Weekly term and relationship validation reports of ASCT+B Tables are available on GitHub^86^.

All data and SOPs are released under Creative Commons Attribution 4.0 International (CC BY 4.0).

## Technical Validation

This section covers comparison to other KGs; growth of the HRA coverage and usage over time; and term additions to Uberon and CL by the HRA effort.

### Comparison to other KGs

We compared the HRA KG with other major biomedical KGs quantitatively (number of nodes, node types, edges, edge types, and size) and qualitatively (date of latest release, technology used, accessibility via SPARQL, need for authentication, presence of API for canned queries, license, and reproducibility), see Table 3. Jupyter Notebook with queries for statistics in support of this comparison is on the companion website at cns-iu.github.io/hra-kg-supporting-information/#comparison-to-other-kgs.

**Table 3:** Key properties of the HRA KG and other KGs.

Property	HRA KG	SPARC KG	Petagraph	UBKG (Data Distillery)	Ubergraph	ORKG
#Nodes	1,543,074	188,146	32,192,544	54,154,737	4,071,817	557,821
#Node types	1,540,507	164,750	3	5	3,942,352	556,706
#Edges	101,720,865	812,969	151,690,690	227,826,739	458,605,464	1,853,694
#Edge types	509	221	1,960	2,260	778	4,217
Size*	30,848 MB	789 MB	138,571 MB	162,895 MB	96,450 MB	437 MB
Date of latest release	Dec 15, 2024	Sept 21, 2024	May 5, 2024	Jan 3, 2025	Jan 13, 2025	Feb 11, 2025
Technology	RDF/SPARQL	RDF/SPARQL	Neo4J	Neo4J	RDF/SPARQL	RDF/SPARQL (Neo4J also available)
Provides SPARQL endpoint	X	X			X	X
No authenticated access needed for queries	X	X			X	X
Has API with canned queries	X	X	X	X	X	X
Data Usage License (strictest listed)	CC BY 4.0	CC BY 4.0	UMLS License	UMLS License	CC BY 4.0	CC0 1.0 Universal**
Data and code full reproducible	X	X	X	X	X	X

^*^RDF-based KGs were converted to N-Quads to get the total uncompressed size for each. To compare sizes, Neo4J KGs were converted to RDF N-Quad format; context and documentation are at cns-iu.github.io/hra-kg-supporting-information/#comparison-to-other-kgs. A Neo4J utility (neo4j.com/labs/apoc/4.1/export/json) was used to export a whole database in the JSON-lines format (jsonlines.org) and convert it to RDF using a JSON-LD context with newline-delimited JSON (ndjsonld, www.npmjs.com/package/ndjsonld).

**Data sourced from Papers With Code, licensed CC BY-SA.

**Fig. 4:**
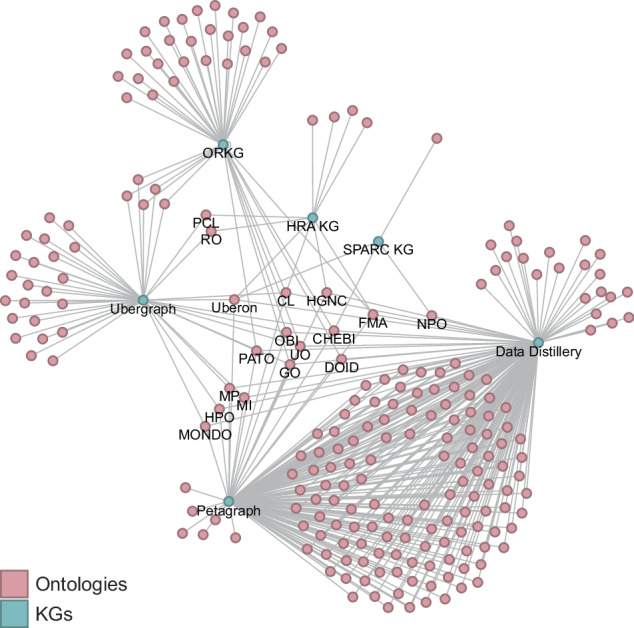
Bimodal network of KGs and the ontologies they ingest and serve. Labels are only shown for nodes with a degree greater than or equal to three, i.e., the KGs themselves and all ontologies shared by at least three KGs. Two ontologies have manually added labels (PCL, RO). Note that Petagraph and Data Distillery ingest many of the same ontologies and are thus very similar. The most shared ontologies across KGs are Uberon, CL, FMA, HGNC, CHEBI, STRING, DOID, UO, PATO, OBI, and GO. The layout was made with Gephi (gephi.org), using the Yifan Hu (Proportional) Algorithm for layout (yifanhu.net/PUB/graph_draw.pdf).

The Stimulating Peripheral Activity to Relieve Conditions (SPARC)^87^ Connectivity Knowledge Base of the Autonomic Nervous System (SCKAN)^88,89^ provides FAIR vocabulary for its multimodal models, data, maps, and simulations across species. It ingests community ontologies across SPARC-relevant domains, such as physiology, anatomy, molecular structures, and experimental design (see Fig. 4), and serves the data via an endpoint at blazegraph.scicrunch.io/blazegraph/sparql. Petagraph^25^ and UBKG were introduced in the **Background & Summary** section. Petagraph and UBKG data were retrieved from the Common Fund Data Ecosystem (CFDE) Data Distillery (dd-kg-ui.cfde.cloud/about). Ubergraph^90^ is a RDF triplestore with a public SPARQL endpoint that makes 39 ontologies from the OBO Foundry^10,11^ available as a pre-computed KG to support ontology browsing and connection verification. Finally, the Open Research Knowledge Graph (ORKG)^91,92^ aims to improve processing of scholarly knowledge via an infrastructure that makes the description of research contributions machine-readable; it uses LLMs to support natural language queries (ask.orkg.org).

The number of nodes and complexity of these KGs ranges from 188k nodes of 165k types to 54 M nodes of 5 types. The size ranges from 437 MB for ORKG to 162GB for UBKG (Data Distillery version).

The UBKG and Petagraph use the Neo4J platform and require a UMLS License (www.nlm.nih.gov/databases/umls.html). ORKG has a CC0 1.0 Universal license. All other KGs are open access with the CC BY 4.0 license (creativecommons.org/licenses/by/4.0/deed.en).

**Note**: One complication when comparing KGs is that data representation is different between RDF-based KGs using RDF/SPARQL and Property Graph (PG)-based KGs using Neo4J. RDF consists of only edges (triples) with subject, predicate, and object. PGs consist of a set of nodes and edges. In RDF, nodes (i.e., resources) are annotated via an edge/triple (e.g., <https://example.com> <http://www.w3.org/2000/01/rdf-schema#label> “Example.com Label”) whereas in a PG, annotations are just part of the node’s data structure. That is, in RDF, edges are used to both annotate nodes and represent relationships between nodes. As a result, comparing raw edge counts between a PG and RDF graph is complicated. To account for this, corresponding SPARQL (for RDF Graphs) and Cypher (for PGs, neo4j.com/docs/cypher-manual/current/introduction) queries were written to retrieve the number of nodes, edges, and edge types that, while not perfect, still gives a sense of their quantitative relationship. A full list of SPARQL and Cypher queries can be found in the Supporting Information at cns-iu.github.io/hra-kg-supporting-information/#comparison-queries.

Fig. 4 shows the bimodal network of the seven KGs (blue nodes) and the 288 ontologies (pink nodes) they import and serve. All but ORKG import Uberon and CL and as a result, they cover the same organs, ASs, and CTs that exist in these ontologies. The HRA KG has 951 additional TEMP-ASs, 221 TEMP-CTs, and 296 TEMP-Biomarkers (genes, proteins, lipids, metabolites, proteoforms) that were identified by human experts as missing and will be added to Uberon, CL, and biomarker ontologies. A SPARQL query to retrieve the number of TEMP entities in the HRA KG as of May 2025 is available on the companion website at cns-iu.github.io/hra-kg-supporting-information/#example-queries.

### Growth of Uberon, CL, and PCL over Time from other Sources

To compare the growth of the HRA KG against other KGs, the number of nodes, edges, and edge types was computed for Uberon, CL, and PCL. To that end, OWL files with release dates were downloaded from GitHub repositories (Uberon^93^, CL^94^, PCL^95^), then queried with a SPARQL query. The resulting CSV file holds the number of nodes, edges, and edge types for Uberon, CL, and PCL by release. All data and code is available in the Supporting Information on GitHub^96^. The CSV file at github.com/cns-iu/hra-kg-supporting-information/blob/main/notebooks/output/other-ontologies-growth.csv shows the growth of these three KGs between 2022 and 2025. Note that Uberon was first started in 2012, but the data available via releases in GitHub only goes back to 2022. The number of edge types is relatively stable, hovering around 100 for all three. The number of nodes and edges has been stable in Uberon and CL but has seen a steep increase for PCL since mid-2024, highlighting the more rapidly changing nature of PCL versus the more stable and established other two ontologies. Since 2022, Uberon/CL/PCL have had at most 15,959/3,122/16,980 nodes, 238,139/30,635/221,269 edges, and 171/58/85 edge types.

### Atlas Coverage

The HRA Dashboard (apps.humanatlas.io/dashboard/data) shows the size and coverage of HRA data; the number and type of HRA usage over time; publication and experimental data linked to the HRA; plus the demographic coverage of HRA authors, tissue donors, and users.

**Fig. 5:**
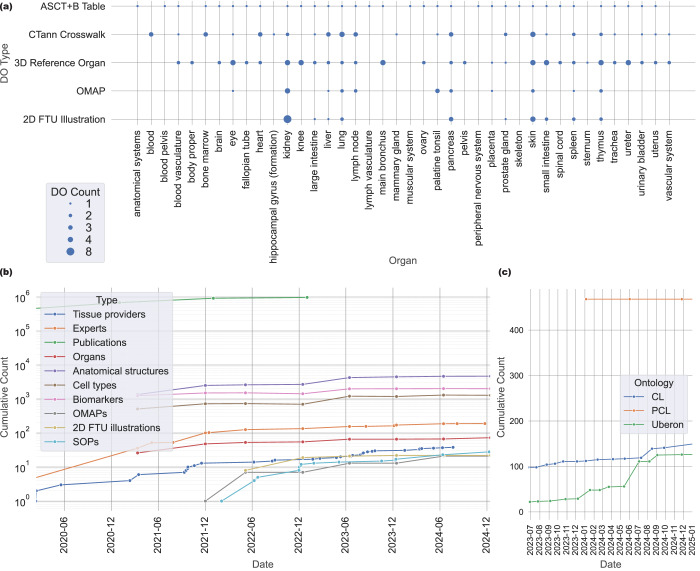
Graphs from the HRA Dashboard showing HRA KG v2.2 growth and coverage: **(a)** DOs per organ, **(b)** HRA KG growth in terms of DOs over time, **(c)** number of terms added from the HRA and other sources to CL, PCL, and Uberon over time.

Fig. 5a shows the number of instances of different DO types. Specifically, HRA KG v2.2 covers 71 organs via 3D *ref-organ* DOs. Note that eye, fallopian tube, kidney, knee, mammary gland, ovary, palatine tonsil, renal pelvis, and ureter have left and right HRA DOs for the same Uberon ID and that exactly five organs are female only (fallopian tube, mammary gland, ovary, placenta, and uterus) while one is male only (prostate). 33 *asct-b* DOs tables exist, where 32 cover organs and one covers anatomical systems. *ctann* crosswalks exist for 23 organs, with 12 organs having crosswalks for more than one CTann annotation tool. In HRA KG v2.2, *omap* DOs exist for 13 organs (including retina and vermiform appendix, which are not shown in Fig. 5a). Finally, *2d-ftu* DOs are present for 10 organs, with the kidney having the most (eight).

Fig. 5b plots HRA growth since HuBMAP started in 2018 (but only shows data since March 2020). The green line shows publications linked to the HRA via the HRAlit^35^ dataset, which captures literature connected to the HRA v1.4 (June 2023). In 2021, more HRA DO types were added (*omap* DOs^34^, later *2d-ftu* DOs^67^), plus a growing library of SOPs detailing HRA construction and usage (humanatlas.io/standard-operating-procedures). As new HRA DOs are published, they are ingested into the HRA KG.

Fig. 5c shows the number of terms that were added to existing ontologies based on SME input via HRA DOs. As of May 2025, the HRA and other atlasing efforts added 162 terms to Uberon, 155 to CL, and 468 to PCL (shown in the line graph are additions between July 2023 and January 2025).

## Usage Notes

Users access the HRA KG via UIs, APIs, and data products on lod.humanatlas.io to answer biomedical questions. A list of all HRA applications that use the HRA KG is provided in Table S3. A list of publications and aliases used throughout HRA applications per HRA DO is provided in Table S6. Exemplary Python code is provided on the companion website at cns-iu.github.io/hra-kg-supporting-information/#basic-usage.

The HRA KG makes it possible to access harmonized, high-quality reference and experimental data in standard data formats. Three widely used queries are detailed: **(1) retrieve AS-CT-B records** from the ASCT+B tables**, (2) get mean B expression values** for CTs across datasets in HRApop, and **(3) query the HRA KG to achieve two types of prediction**s: cell type populations given a spatial origin (3D extraction site), and spatial origin (3D extraction site and registration corridor) given a cell type population, see HRA user stories 1-2 in a related publication^2^. Code for queries and advanced usage examples are presented on GitHub^97^. An overview of HRA user stories and how the HRA KG supports them is provided in Table S7.

To simplify HRA KG usage, the grlc.io service was implemented to make a set of canned SPARQL queries available as RESTful web requests. Internally, the service creates an OpenAPI specification (swagger.io/specification) that advertises the available queries. A user-friendly interface to these queries is provided at apps.humanatlas.io/api/grlc/. Annotated screenshots of the query interface with instructions on how to run the queries and download the resulting data is available on the companion website at cns-iu.github.io/hra-kg-supporting-information/#how-to-run-queries-via-our-openapi-spec. This deployment was inspired by the PubMed Medical Subject Headings (MeSH) SPARQL Explorer at id.nlm.nih.gov/mesh/query.

The HRA KG can also be used in coordination with relational databases. With initial SPARQL queries into the KG, users can get data as a long table. Then, a database system like DuckDB (duckdb.org) can be used to aggregate the KG data with SQL features that perform complex window functions and aggregations on it. That is, users can index the HRA KG data and save it in a relational database and use it for different purposes.

## Supplementary information

Supplementary Information

## Data Availability

All the code used to construct and deploy the HRA KG v2.2 is available on GitHub^46^ and URLs are provided in Table S4. Documentation, including an additional overview of HRA KG construction code, is provided in the Supporting Information repository for this paper on GitHub^97^. Documentation with annotated screenshots to show how to run pre-made SPARQL queries against the HRA KG via grlc.io is at cns-iu.github.io/hra-kg-supporting-information/#how-to-run-queries-via-our-openapi-spec. All code was released under the MIT License.

## Supplementary information

Supplementary material is available at 10.1038/s41597-025-05183-6.
