# Supplementary material for: Construction, Deployment, and Usage of the Human Reference Atlas Knowledge Graph
Source: Sci Data. 2025 Jul 1;12:1100. doi: 10.1038/s41597-025-05183-6 (PMC12218032; doi:10.1038/s41597-025-05183-6)
Supplement: Supplementary file 1 — Supplementary Information [file 41597_2025_5183_MOESM1_ESM.pdf]

Supplementary Information

Construction, Deployment, and Usage of the Human Reference Atlas Knowledge Graph

Andreas Bueckle[1*], Bruce W. Herr II[1*], Josef Hardi[2], Ellen M. Quardokus[1], Mark A. Musen[2], Katy Börner[1*]

[1] Department of Intelligent Systems Engineering, Luddy School of Informatics, Computing, and Engineering, Indiana University, Bloomington, IN, USA
[2] Stanford Center for Biomedical Informatics Research, Stanford University, Stanford, CA, USA
* Corresponding authors
abueckle@iu.edu
bherr@iu.edu
katy@iu.edu

Supplementary Figures


```yaml
data:
  cell_marker_descriptors:
   - id: HRA:R15-desc
     label: Cell marker descriptor for Proximal Tubule  Epithelial Cell
     type_of:
      - CellMarkerDescriptor
     primary_anatomical_structure: UBERON:0004134
     primary_cell_type: CL:0002306
     biomarker_set:
      - HGNC:2548
      - HGNC:6694
      - HGNC:10916
      - HGNC:417
      - HGNC:4175
     references:
       - https://www.doi.org/10.1038/s41467-019-10861-2
       - https://www.doi.org/10.1172/jci.insight.13326
       - https://www.doi.org/10.1038/s41586-020-2941-1
       - https://www.doi.org/10.1073/pnas.2005477117
       - https://www.doi.org/10.1681/ASN.2020010071
     derived_from: https://lod.humanatlas.io/asct-b/kidney/v1.5
```


Figure S1. Excerpt of a normalized structure from the ASCT+B table for the kidney, with a focus on *cell_marker_descriptors*, where primary CT, primary anatomical location, and associated characterizing Bs are detailed, and where references to support the claims regarding the CTs and its Bs are provided. An example in YAML is provided at github.com/cns-iu/hra-kg-supporting-information/blob/main/docs/intermediary_format.yaml.

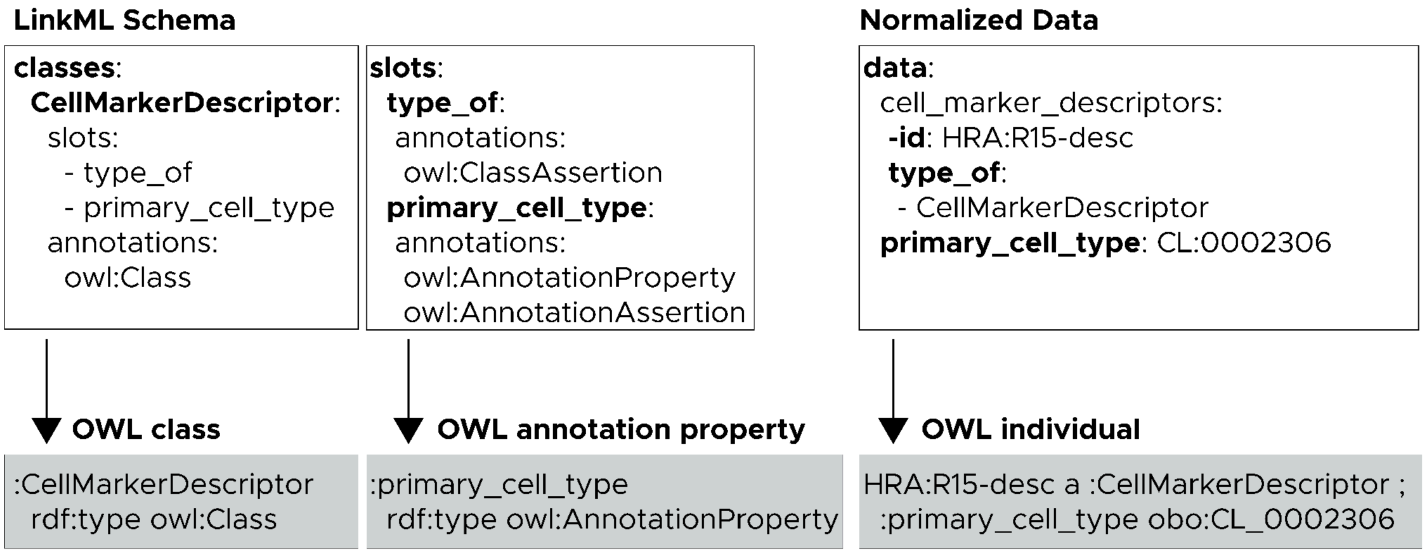

Figure S2. Excerpt of a LinkML schema (represented by the two boxes on the left) and the corresponding acquired data (the box on the right). Arrows illustrate the transformation of the input text into OWL constructs at the bottom. In the schema boxes, classes and slots are directly mapped to OWL classes and OWL properties as specified in the *annotations* field. In the data box, each data item pair is translated into an OWL assertion statement. For example, the data pair *type_of: CellMarkerDescriptor* generates a class assertion that indicates the data object belongs to the CellMarkerDescriptor class. Similarly, the data pair *primary_cell_type: CL:0002306* produces an annotation assertion that tells the same data object identifies the epithelial cell in the proximal tubule of the kidney (CL:0002306) as the primary CT.

Supplemental Tables

Table S1. Graphs of the HRA KG (lod.humanatlas.io/graph).

Name	Description	Link
2d-ftu-illustrations	Contains 2D assets for FTUs.	https://lod.humanatlas.io/graph/2d-ftu-illustrations
ccf	Graph representation of the CCF.OWL dataset.	https://lod.humanatlas.io/graph/ccf
ctann-crosswalks	Graph representation of CTann crosswalks for HRApop (Azimuth[1], CellTypist[2,3] popV[4]).	https://lod.humanatlas.io/graph/ctann-crosswalks
ctpop	(Deprecated) version of the hra-pop *graph*, see below.	https://lod.humanatlas.io/graph/ctpop
ds-graphs-enrichments	Enriched graph of all public dataset graphs for the HRA.	https://lod.humanatlas.io/graph/ds-graphs-enrichments
hra-ccf-body	Graph representation of the 3D Reference Objects for human male and female bodies.	https://lod.humanatlas.io/graph/hra-ccf-body
hra-ccf-patches	Graph that provides placements (patches) to convert old 3D Reference Object registrations to newer versions for reference organs that had a different shape, placement, or size in earlier versions.	https://lod.humanatlas.io/graph/hra-ccf-patches
hra-pop	Contains donor and sample metadata as well as 3D extraction sites and cell summaries for 619 ATLAS datasets used in HRA construction (as of v0.11.1). Code to compute mean B expression values per cell per dataset is at https://github.com/hubmapconsortium/hra-workflows/blob/3dfea9ce19138c930dec92024496c4814a290f12/containers/gene-expression/context/main.py#L108-L151<br><br>Exemplarily, the lookup table between Ensembl Release 111[5] (https://www.ensembl.org/index.html) and HGNC v2023-09-18[6] (https://www.genenames.org) for CellTypist is at https://github.com/hubmapconsortium/hra-workflows/blame/main/containers/celltypist/context/ensemble-lookup.csv	https://lod.humanatlas.io/graph/hra-pop
HRAlit (currently served as a relational database)	Links the HRA DOs to publications, experts, experimental datasets, and funded projects[7] to identify relevant papers, subject matter experts (SMEs), alignment with existing ontologies, and funding trends to HRA construction and usage. Code is available on GitHub[8].	N/A

Table S2. Collections of the HRA KG (lod.humanatlas.io/collection). All structures are built using standardized terms for labels, which are stored in the specialized CCF vocabulary, see CCF Ontology in **Box 1**.

**Name**	**Description**	**Link**
ds-graphs	Consolidates experiment-result datasets (*ds-graph*) into a single graph data structure. Contains processed graphs of datasets including donor and sample metadata from different consortia and tissue providers registered to HRA via 3D extraction sites, available at https://lod.humanatlas.io/ds-graph.	https://lod.humanatlas.io/collection/ds-graphs
hra	Includes HRA Digital Object (DO) types with a DOI. As of HRA v2.2, those are *2d-ftu*, *asct-b*, *ctann, omap*, *ref-organ*, and *vascular-geometry* DO types. Other DO types can be added in the future once they have DOIs.	https://lod.humanatlas.io/collection/hra
hra-api	Centers on *asct-b* and associated 3D models (*ref-organ, landmark*) used in various HRA applications for visualizing and organizing reference data.	https://lod.humanatlas.io/collection/hra-api
hra-ols	HRA collection subset that focuses solely on AS, CTs, and Bs (*asct-b* only). It is used by OLS (https://www.ebi.ac.uk/ols4) for validation. The source for the files on the LOD server are available on GitHub[9].	https://lod.humanatlas.io/collection/hra-ols

Table S3. Most HRA applications use the HRA KG as their backend database. Also listed are external applications that use the HRA KG.

Name	Description	URL
HRA Portal	Provides the landing page and additional web pages for exploring HRA-related resources. Some data statistics on the page are aggregated from the HRA KG via SPARQL queries.	https://humanatlas.io
RUI	Allows users to register new tissue samples and related datasets into the human reference atlas[10]. The "parts" hierarchy on the navigation panel and the 3D human model at the center of the page are generated from the HRA KG via HRA API queries.	https://apps.humanatlas.io/rui
EUI	Enables users to explore and query the HRA KG interactively[10]. All visualizations, data facets, statistics, and data browsers are generated through real-time querying of the HRA KG.	https://apps.humanatlas.io/eui
ASCT+B Reporter	Enables users to inspect ASCT+B Tables as tree visualizations[10] and supports OMAP comparison to each other and to ASCT+B tables. Uses the HRA KG to serve processed and published ASCT+B Tables to the website.	https://apps.humanatlas.io/asctb-reporter
FTU Explorer	Facilitates interactive exploration of FTUs within the HRA. The HRA KG provides the *2d-ftu* DOs that the FTU Explorer displays. Datasets and cell summaries registered to these FTUs are currently separate but will be available in the HRA KG as *ds-graph* DOs in the future.	https://humanatlas.io/2d-ftu-illustrations
HRA Dashboard	Offers a summary and visual representation of data statistics and trends within the HRA knowledge graph. Data statistics and charts are generated by aggregating data from the HRA KG.	https://apps.humanatlas.io/dashboard
HRA API endpoints	Provides programmatic access to the HRA KG, enabling integration with other applications and services. A SPARQL endpoint for the HRA API allows users to write their own SPARQL queries. Canned queries are available at https://apps.humanatlas.io/api/grlc/.	API documentation: https://humanatlas.io/api
HRA Organ Gallery	Enables an immersive view of the HRA by showing 71 reference organs and 1,100+ tissue blocks alongside cell type populations in VR[11,12].	https://humanatlas.io/hra-organ-gallery
Cell Distance Explorer	Allows distance visualizations between cells in 2D and 3D. Features histograms and violin graphs for vascular distances by CT. Does not use HRA KG but will use it for crosswalking CTs to CL and PCL in the future.	https://apps.humanatlas.io/cde
**In Data Portals**		
HuBMAP Data Portal	Same as EUI above but only showing HuBMAP data.	https://portal.hubmapconsortium.org/ccf-eui
SenNet Data Portal	Same as EUI above but only showing SenNet data.	https://data.sennetconsortium.org/ccf-eui
GTEx Portal	Same as EUI above but only showing GTEx data.	https://gtexportal.org/home/eui
KPMP Tissue Atlas	Same as EUI above but only showing KPMP data.	https://apps.humanatlas.io/eui/kpmp.html

Table S4. An overview of all GitHub repositories used to construct, deploy, and use the HRA KG.

Name	Description	URL
**Code**		
hra-kg	HRA Knowledge Graph	GitHub[13]
hra-api	HRA API	GitHub[14–16]
hra-do-processor	HRA DO Processor	GitHub[17]
ccf-grlc	Repository of canned SPARQL queries that can be run like a REST API endpoint via https://grlc.io.	GitHub[18]
hra-ubkg-exporter	A command line interface (CLI) for exporting a subset of the HRA KG to Unified Biomedical Knowledge Graph (UBKG) format.	GitHub[19]
hra-kg-releases/hra-kg.v2.2.tar.xz	Whole HRA KG, ~5.1GB.	Zenodo[20]<br><br>HRA CDN: https://cdn.humanatlas.io/hra-kg-releases/hra-kg.v2.2.tar.xz
**APIs and Documentation**		
HRA KG SPARQL endpoint	SPARQL endpoint of the HRA KG	https://lod.humanatlas.io/sparql
HRA API	UI with documentation for all HRA API endpoints and routes	https://apps.humanatlas.io/api
HRA API SPARQL queries	Canned SPARQL queries via grlc.io on HRA API	https://apps.humanatlas.io/api/grlc/

Table S5. List of LinkML schemas in the HRA KG v2.2.

Schema Description	Link
LinkML schema for *asct-b* DOs	https://github.com/hubmapconsortium/hra-do-processor/blob/main/schemas/src/digital-objects/asct-b.yaml
LinkML schema for *ref-organ* DOs	https://github.com/hubmapconsortium/hra-do-processor/blob/main/schemas/src/digital-objects/ref-organ.yaml
LinkML schema for *2d-ftu* DOs	https://github.com/hubmapconsortium/hra-do-processor/blob/main/schemas/src/digital-objects/2d-ftu.yaml
LinkML schema for *landmark* DOs	https://github.com/hubmapconsortium/hra-do-processor/blob/main/schemas/src/digital-objects/landmark.yaml
LinkML schema for *omap* DOs	https://github.com/hubmapconsortium/hra-do-processor/blob/main/schemas/src/digital-objects/omap.yaml
LinkML schema for *ds-graph* DOs	https://github.com/hubmapconsortium/hra-do-processor/blob/main/schemas/src/digital-objects/ds-graph.yaml
LinkML schema for *graph* DOs	https://github.com/hubmapconsortium/hra-do-processor/blob/main/schemas/src/digital-objects/graph.yaml
LinkML schema for *vocab* DOs	https://github.com/hubmapconsortium/hra-do-processor/blob/main/schemas/src/digital-objects/vocab.yaml
LinkML schema for *collection* DOs	https://github.com/hubmapconsortium/hra-do-processor/blob/main/schemas/src/digital-objects/collection.yaml
LinkML schema for cell summaries	https://github.com/hubmapconsortium/hra-do-processor/blob/main/schemas/src/modules/cell-summary.yaml
LinkML schema for spatial data	https://github.com/hubmapconsortium/hra-do-processor/blob/main/schemas/src/modules/spatial.yaml
LinkML schema for spatial collision data	https://github.com/hubmapconsortium/hra-do-processor/blob/main/schemas/src/modules/collision.yaml
LinkML schema for spatial corridor data	https://github.com/hubmapconsortium/hra-do-processor/blob/main/schemas/src/modules/corridor.yaml
LinkML schema for assay dataset metadata	https://github.com/hubmapconsortium/hra-do-processor/blob/main/schemas/src/modules/dataset.yaml
LinkML schema for donor metadata	https://github.com/hubmapconsortium/hra-do-processor/blob/main/schemas/src/modules/donor.yaml
LinkML schema for sample metadata	https://github.com/hubmapconsortium/hra-do-processor/blob/main/schemas/src/modules/sample.yaml
LinkML schema for defining entities	https://github.com/hubmapconsortium/hra-do-processor/blob/main/schemas/src/shared/entity-base.yaml
LinkML schema for defining instances	https://github.com/hubmapconsortium/hra-do-processor/blob/main/schemas/src/shared/instance-base.yaml

LinkML schema for defining DO metadata	https://github.com/hubmapconsortium/hra-do-processor/blob/main/schemas/src/shared/metadata-base.yaml

Table S6. Publications on HRA DOs plus aliases used throughout HRA publications and applications.

HRA DO Type	Aliases	Publication Title	DOI
2d-ftu	- Functional Tissue Units<br>- Functional Tissue Units (FTUs)<br>- FTUs	Functional Tissue Units in the Human Reference Atlas	https://doi.org/10.1101/2023.10.16.562593
asct-b	- Anatomical Structures, Cell Types, and Biomarker Tables<br>- Anatomical Structures, Cell Types, and Biomarker Tables (ASCT+B) Tables<br>- ASCT+B Tables	Anatomical structures, cell types and biomarkers of the Human Reference Atlas	https://www.nature.com/articles/s41556-021-00788-6
		Anatomical structures, cell types, and biomarkers of the healthy human blood vasculature	https://www.nature.com/articles/s41597-023-02018-0
ctann	- Cell Type Annotations<br>- CTann		
landmark	- Landmark Organs<br>- Landmark Anatomical Structures		
millitome	- Millitome		
omap	- Organ Mapping Antibody Panels<br>- Organ Mapping Antibody Panels (OMAPs)<br>- OMAPs<br>- Organ Mapping Antibody Panel Tables	Organ Mapping Antibody Panels: a community resource for standardized multiplexed tissue imaging	https://www.nature.com/articles/s41592-023-01846-7
ref-organ	- 3D Reference Object<br>- 3D Reference Organ	Tissue registration and exploration user interfaces in support of a human reference atlas	https://www.nature.com/articles/s42003-022-03644-x
schema	- Schema		
vascular-geometry		Anatomical structures, cell types, and biomarkers of the healthy human blood vasculature	https://www.nature.com/articles/s41597-023-02018-0
		Considerations for Using the Vasculature as a Coordinate System to Map All the Cells in the Human Body	https://doi.org/10.3389/fcvm.2020.00029
vocab		Specimen, biological structure, and spatial ontologies in support of a Human Reference Atlas	https://www.nature.com/articles/s41597-023-01993-8

Table S7. HRA User Stories and how the HRA KG supports them.

HRA User Story	Value Added by HRA KG	HRA KG Sample Queries
**US#1.** Predict cell type populations	Use the HRA KG to access the hra-pop *graph* (https://lod.humanatlas.io/graph/hra-pop/latest) with cell type populations for AS, datasets, and extraction sites to improve the accuracy of annotations for sc-transcriptomics and sc-proteomics datasets. In the future, the HRA KG could be used to store metadata for millions of cells individually.	/as-weighted-cell-summaries: https://github.com/x-atlas-consortia/hra-api/blob/main/src/library/hra-pop/queries/as-weighted-cell-summaries.rq<br><br>Accessible via HRA API: https://apps.humanatlas.io/api/hra-pop/rui-location-cell-summary
**US#2.** Predict spatial origin of tissue samples	Use the cell type populations from the hra-pop *graph* mentioned above to predict the 3D location of datasets with unknown spatial origin.	/select-cell-summaries: https://github.com/x-atlas-consortia/hra-api/blob/main/src/library/hra-pop/queries/select-cell-summaries.rq<br><br>Accessible via HRA API: https://apps.humanatlas.io/api/hra-pop/cell-summary-report<br><br>/supported-organs: https://github.com/x-atlas-consortia/hra-api/blob/main/src/library/hra-pop/queries/supported-organs.rq<br><br>/supported-reference-organs: https://github.com/x-atlas-consortia/hra-api/blob/main/src/library/hra-pop/queries/supported-reference-organs.rq<br><br>/supported-tools: https://github.com/x-atlas-consortia/hra-api/blob/main/src/library/hra-pop/queries/supported-tools.rq
**US#3.** Compare reference tissue with aging/diseased tissue	Use the EUI to examine AS, CTs, and Bs. Via the HRA API, the RUI runs SPARQL queries of the HRA KG to retrieve the AS partonomy, CT typology, and Bs.	/tissue-blocks: https://apps.humanatlas.io/api/#get-/v1/tissue-blocks (then user parameters to get datasets by age (range), BMI (range), sex<br><br>Accessible via HRA API: https://apps.humanatlas.io/api/#get-/v1/tissue-blocks (then user parameters to get datasets by age (range), BMI (range), sex<br><br>/scene: https://github.com/x-atlas-consortia/hra-api/blob/main/src/library/v1/queries/scene.rq<br>Note: #{{FILTER}} gets replaced with filters documented on HRA API endpoint at https://apps.humanatlas.io/api/#get-/v1/scene to get datasets by age (range), BMI (range), sex, etc.
**US#4.** Compare reference FTUs with aging/diseased FTUs	Use the HRA KG to retrieve 2D illustrations of 22 FTUs in the FTU Explorer (https://apps.humanatlas.io/ftu-explorer/#)	/ftu-parts: https://apps.humanatlas.io/api/grlc/hra.html#get-/ftu-parts

		/2d-ftu-illustrations (DO) https://lod.humanatlas.io/graph/2d-ftu-illustrations
**US#5.** Provide cell distance distribution visualizations	Use the Cell Distance Explorer to visualize distance distributions between different cells and CT in 2D or 3D sc-proteomics tissue. In the future, the HRA KG will be used to crosswalk CT labels to CL (https://obofoundry.org/ontology/cl.html)[21] to add automated grouping features. Cell distances could also be stored in the HRA KG.	No sample queries yet, but support is possible for adding comparisons to other datasets or simpler color scheme via CL once crosswalks exist
**US#6.** Develop lightweight atlas components	Use the HRA KG to enable HRA web components to improve data access and analysis outside of the HRA applications ecosystem (https://apps.humanatlas.io/us6). More information is provided under Results > Using the HRA KG > HRA Applications.	Components use HRA KG, e.g., EUI component uses HRA KG via HRA API, same for RUI and FTU Explorer (see US#3 and 4)
**US#7.** Implement dashboard for HRA	Explore usage statistics of atlas data and code to check HRA growth and outreach over time. SPARQL queries to the HRA KG serve stats on experimental data and all HRA DO types	/digital-objects-per-organ: https://github.com/x-atlas-consortia/hra-dashboard-data/blob/main/queries/sparql/data/digital-objects-per-organ.rq<br><br>/as-per-organ: https://github.com/x-atlas-consortia/hra-dashboard-data/blob/main/queries/sparql/humanatlas.io/as-per-organ.rq<br><br>hra-growth.atlas.rq https://github.com/x-atlas-consortia/hra-dashboard-data/blob/main/queries/sparql/data/hra-growth.atlas.rq

References

1. Hao, Y. *et al.* Integrated analysis of multimodal single-cell data. *Cell* **184**, 3573-3587.e29 (2021).
2. Domínguez Conde, C. *et al.* Cross-tissue immune cell analysis reveals tissue-specific features in humans. *Science* **376**, eabl5197 (2022).
3. Xu, C. *et al.* Automatic cell-type harmonization and integration across Human Cell Atlas datasets. *Cell* **186**, 5876-5891.e20 (2023).
4. Ergen, C. *et al.* Consensus prediction of cell type labels in single-cell data with popV. *Nat. Genet.* (2024) doi:10.1038/s41588-024-01993-3.
5. Seal, R. L. *et al.* Genenames.org: the HGNC resources in 2023. *Nucleic Acids Res.* **51**, D1003–D1009 (2023).
6. Martin, F. J. *et al.* Ensembl 2023. *Nucleic Acids Res.* **51**, D933–D941 (2023).
7. Kong, Y. & Börner, K. Publication, funding, and experimental data in support of Human Reference Atlas construction and usage. *Sci. Data* **11**, 574 (2024).
8. Cyberinfrastructure for Network Science Center. x-atlas-consortia/hra-lit: Publication, funding, and experimental data in support of Human Reference Atlas construction and usage (HRAlit). https://github.com/x-atlas-consortia/hra-lit (2025).
9. HuBMAP Consortium. hubmapconsortium/3d-hra-ref-object-validation: HRA 3D Reference Object Validation Reports. https://github.com/hubmapconsortium/3d-hra-ref-object-validation (2024).
10. Börner, K. *et al.* Tissue registration and exploration user interfaces in support of a human reference atlas. *Commun. Biol.* **5**, 1369 (2022).
11. Bueckle, A. *et al.* The HRA Organ Gallery affords immersive superpowers for building and exploring the Human Reference Atlas with virtual reality. *Front. Bioinforma.* **3**, (2023).
12. Cyberinfrastructure for Network Science Center. HRA Organ Gallery on Horizon Store. *Oculus* https://www.meta.com/experiences/quest/5696814507101529/ (2024).
13. HuBMAP Consortium. hubmapconsortium/hra-kg: Human Reference Atlas Knowledge Graph. https://github.com/hubmapconsortium/hra-kg (2025).
14. HuBMAP Consortium. x-atlas-consortia/hra-api: Human Reference Atlas API. https://github.com/x-atlas-consortia/hra-api (2025).
15. Cyberinfrastructure for Network Science Center. HRA-API Documentation. https://apps.humanatlas.io/api/ (2024).
16. Cyberinfrastructure for Network Science Center. hra-api/notebooks at main · x-atlas-consortia/hra-api: HRA-API: Human Reference Atlas API. *GitHub* https://github.com/x-atlas-consortia/hra-api/tree/main/notebooks (2025).
17. HuBMAP Consortium. hubmapconsortium/hra-do-processor: HRA Digital Objects Processor. https://github.com/hubmapconsortium/hra-do-processor (2025).
18. Cyberinfrastructure for Network Science Center. hubmapconsortium/ccf-grlc. https://github.com/hubmapconsortium/ccf-grlc (2024).
19. HuBMAP Consortium. x-atlas-consortia/hra-ubkg-exporter: A CLI for exporting a subset of the HRA Knowledge Graph to UBKG format. https://github.com/x-atlas-consortia/hra-ubkg-exporter (2024).
20. Bueckle, A., Herr, B. & Börner, K. HRA Knowledge Graph v2.2. Zenodo https://doi.org/10.5281/zenodo.15323983 (2025).
21. Diehl, A. D. *et al.* The Cell Ontology 2016: enhanced content, modularization, and ontology interoperability. *J. Biomed. Semant.* **7**, 44 (2016).
